# Creation and Analysis of a Respiratory Sensor Using the Screen-Printing Method and the Arduino Platform

**DOI:** 10.3390/s23042315

**Published:** 2023-02-19

**Authors:** Jarosław Wojciechowski, Ewa Skrzetuska

**Affiliations:** Faculty of Material Technologies and Textile Design, Institute of Material Science of Textiles and Polymer Composites, Lodz University of Technology, 116 Zeromskiego Street, 90-924 Lodz, Poland

**Keywords:** textile actuator, T-shirt, textronic, monitor, microcontroller, Arduino, human body, carbon nanotubes, screen printing, printing composition, textiles

## Abstract

The aim of this paper is to present novel highly sensitive and stretchable strain sensors using data analysis to report on human live parameters using the Arduino embedded system as a proof of concept in developing new and innovative solutions for health care. The article introduces the solution of textile sensor origination with electrical resistance measurement using the mobile Arduino microcontroller in the designed/elaborated textile printed sensor. The textile sensor was developed by the screen printing technique based on the water dispersion of carbon nanotubes during printing composition. By stretching and squeezing the T-shirt during breathing, the electrical resistances of the printed sensor were changed. The measured resistance corresponded to the number of breaths of the person wearing the T-shirt. The microcontroller calculated the number of breaths as a number of electrical resistance peaks, which then led to monitoring human live parameters.

## 1. Introduction

The growing popularity of textronic systems has been closely related to progress in the miniaturization of electronics and the development of textiles and textile technologies. They enable, among other things, the creation of new solutions in the field of measuring vital functions, which are an important element of each of the aforementioned areas. Such systems embedded in the structure of clothing are supposed to fulfill their function without disturbing the usable comfort (physical, physiological and mental balance) of the person wearing it. This is possible thanks to the use of the characteristic properties of some raw materials from which textile products are made, i.e., piezo-electric, electrostrictive and shape memory properties. Materials using these features are called intelligent, and they combine the functions of both a sensor and an actuator. Their properties change under the influence of an external stimulus, which may be, for example, a mechanical stimulus or an electrical impulse [1].

In the case of vital signs sensors, textronic systems are most often based on piezoelectric materials, in which an electrical charge is generated as a result of changes in the dimensions of the material caused by the movement of the user’s chest. However, the use of printing techniques in the creation of deformation sensors is more often considered. Prints made on a knitted substrate with the use of pastes or inks based on, for example, carbon nanotubes, can bring about a number of new possibilities. Potentially, printed sensors could be more convenient to use, flexible, non-restrictive of movements and, at the same time, more sensitive and give adequate measurement results [2]. Each system, the primary task of which is to take measurements, must have a sensor in its structure. It is an element directly responsible for the identification of external stimuli; for example, the recognition of the value of a measured physical quantity or the detection of a specific signal. In most cases, the sensor systems used in technical sciences are based on easy-to-process electrical quantities, such as an electrical resistance, current and voltage [3]. Commercially available textronic measuring systems operate most often on the basis of the piezoelectric properties of the intelligent materials used in their construction. The piezoelectric phenomenon occurs in solids characterized by a specific ordering of the atoms that build them. This consists of the formation of electrical induction on the surface of these bodies as a result of their mechanical deformation. There is a linear relationship between the generated electric field and the stress applied to a solid [4]. There are at least a few products on the market that allow for convenient and non-complicated monitoring of the user’s vital functions. These systems are built using smart materials.

The Textronics^®^ company offers NuMetrex^®^ sportswear equipped with Textro-Sensors^®^ textile sensors measuring the respiratory rhythm and heart rate. The sensors are based on flexible conductive yarns built directly into the garment. Changes in the dimensions of the product, caused by the movement of the chest, generate an electrical impulse. This signal is then sent to a small transmitter placed on the outer side of the garment, which can be synchronized with a user-selected device displaying information about the tested rhythm, e.g., a sports watch or a smart phone. The company assures that their systems are based entirely on textile products, thus using no additional wires or electrodes [1,5]. Another example of a textronic solution is the system developed as part of the “Wearable Health Care System” project. Its aim is to integrate computer techniques, intelligent sensors and portable and telecommunication devices in textile products. The proposed system is intended to support patients during rehabilitation or entities working in extreme environmental conditions, ensuring continuous monitoring [6].

There have been studies that have produced piezoresistive pressure sensors based on MoSe_2_ nanosheets. High-performance pressure sensors to detect wrist pulses require a highly sensitive pressure sensor with an extremely low detection limit. To meet the demand for a high-performance device, a piezoresistive sensor functionalized by MoSe_2_ or Wse_2_ nanosheets were fabricated, which showed high-pressure sensitivity in a pressure range of 0.001–100 kPa [7,8].

Another solution is a flexible paper-based sensor functionalized by 2D-SnSe_2_ nanosheets for multifunctional sensing purposes. 2D-SnSe_2_ nanosheets have been synthesized by a high yield liquid phase exfoliation technique. A multifunctional sensor made using Whatman filter paper was used in a pressure range from 2 to 100 kPa. The sensor exhibits excellent stability and repeatability with a high responsiveness of 611% and a sensitivity of 1.79 kPa^−1^. The sensor showed excellent stability up to 5000 charge cycles. The response time of the pressure sensors is 100 ms, indicating the optimal sensor structure for future electronics. The sensor shows excellent potential for online monitoring of human respiration at 3.2–3.5 s/breath [9].

The development of highly sensitive and stretchable pressure sensors is an emerging technological goal in the field of electronic skin and wearable health monitors; these applications require sensors that are sensitive enough to electronic skin or record a human pulse wave while facilitating conformal attachment to a soft, curved surface. To achieve a high degree of sensitivity in the low-pressure regime (<10 kPa), unconventional technologies, such as microstructure polydimethylsiloxane (PDMS), a stretchable resistive pressure sensor, is achieved by coating a compressible substrate with a highly stretchable electrode. The substrate contains an array of microscale pyramidal features, and the electrode comprises a polymer composite. When the pressure-induced geometrical change experienced by the electrode is maximized at 40% elongation, a sensitivity of 10.3 kPa^−1^ is achieved [10].

The current findings show a huge advance over previous reports in portable and large-area smart electronic devices [10,11,12,13,14,15].

For many years, printing techniques have been used to apply decorative patterns to textiles. Recently, however, they are increasingly often used to provide products with other functions of practical application.

The simplest and probably the most used technique in this case is film printing. It consists of applying a printing composition in the form of a printing screen placed on the surface of the textile product. It is a woven mesh made of metal or synthetic material, the pattern of which is given by closing selected meshes with special emulsions [11]. 

The presently existing screen-printing machines allow printing not only on flat surfaces, but also on cylindrical, oval and round surfaces. In the case of this work, the desired property of the intelligent material was the increased ability to conduct electrical impulses. This effect was achieved thanks to the use of conductive pastes as printing compositions. These pastes contain electrically conductive compounds in the form of, for example, nanoparticles of metals such as silver, gold and copper, or carbon compounds such as carbon nanotubes or graphite [2,12,13].

The development of highly sensitive and stretchable strain sensors is a very important element aimed at developing new and innovative solutions for health care. Carbon structures and electrically conductive polymers are becoming increasingly popular. This is due to the fact that they have extremely high chemical and environmental stability and good Van der Waals bonding, which makes them promising materials for durable and sustainable pressure applications in the field of electronic skin monitoring and wearable devices.

Carbon nanotubes are cylindrical, hollow structures composed of one or more layers of carbon. Single-walled structures (SWCNT) are characterized by diameters of several nanometers and many times longer lengths, while multi-walled structures (MWCNT) may have a cross-section with dimensions exceeding 25 nm and a length of 1 μm [16]. Another polymer for which the application of strain sensors is tested is polypyrrole (PPy). Polypyrrole is an organic chemical compound that, like carbon nanotubes, has electrically conductive properties. Additionally, it is durable, highly resistant to weather conditions, and is characterized by biocompatibility [17,18]. These set of characteristics determine polypyrrole for potential use in printed strain sensors. Among the conductive materials, one can also find poly (3,4-ethylene-1,4-dioxythiophene), otherwise known as PEDOT. This compound is known to be one of the most durable conductive polymers. It is characterized by high chemical stability. It is insoluble in water, but it is possible to form an aqueous suspension after combining the PEDOT compound with the PSS polymer [19].

In the case of vital signs sensors, textronic systems are most often based on piezoresistive materials, in which a change in the electrical resistance of the material occurs under the influence of a mechanical force, caused, for example, by the movement of the user’s chest. The use of printing techniques in the creation of deformation sensors is one of the methods increasingly considered in aspects of clothing functionalization. Prints made on a knitted substrate using pastes or inks, for example, based on carbon nanotubes, can present a number of new possibilities. Potentially, printed sensors are more convenient to use, flexible, do not confine movement and, at the same time, are more sensitive and provide adequate measurement results [20].

The choice of a pioneering topic, not only in Poland, but also on a global scale, is the result of analysis of the directions for the development of the textile industry, aimed at, among others goals, creating and the implementing smart clothing. The tasks to be performed by the new intelligent textile materials are to act in systems sensory devices monitoring; e.g., human life activities or detecting threats in the form of chemical compounds in liquids and volatile forms that are dangerous to health and life. Non-invasive or minimally invasive physiological monitoring devices are of great importance for defense purposes and in sports applications. The integration of sensors and biosensors directly into clothing is important, among others reasons, for the development of health care, threat analysis in the case of uniformed services, as well as in monitoring the level of effort of athletes. Smart clothing is primarily the integration of electronics with clothing [21,22]. The existing solutions in this field are aimed toward replacing conventional electronic sensors based on stiff and uncomfortable materials with detectors based on the knowledge gained in the field of designing flexible electronics. The abovementioned three aspects of wearing comfort are equally important. In their absence, it is impossible for the product to give a person the required sense of comfort and balance. It should be noted, however, that this is strictly an individual matter. Preferences as to the raw material, stiffness or softness of clothing may significantly differ, even in the case of people in identical climatic conditions. The aesthetic preferences of individual users are specific aspects that cannot be objectively assessed.

The manufacturing of textronic systems is not an easy task. In order to meet certain properties of the garment, it is necessary to pay attention to a number of factors. When creating a design of clothes, one cannot forget about the requirements that accompany electronic products (including accuracy, measuring range), the behavior of selected textile products (including low weight, flexibility), as well as the applicable principles of materials science and automation. The proper selection of textiles and electronic systems, as well as their mutual integration, is a big challenge for scientists. With the advancement of technological development, the quality of manufactured systems has improved. Textronic products are mainly created using everyday clothes by combining them with a miniaturized electronic system, sensors and a power supply system.

The creation of the textronic system is possible due to the use of sensors. The characteristic properties of some raw materials from which textile products are made include piezoelectric and electrostatic properties, as well as shape memory. Materials using these features are called intelligent, and they combine the functions of both the sensor and the activator.

The most common sensors are sensors that provide information in one of the electrical quantities, such as voltage, current and electrical resistance. This is due to the fact that electric current is a signal that is easily amplified, transmitted over long distances, further processed using digital techniques and computers and saved. Their properties change under the influence of an external stimulus, which may be, for example, a mechanical stimulus or an electrical impulse. 

Several research studies [12,13,23,24,25] have been conducted at the Institute of Material Science of Textiles and Polymer Composites in Lodz, Poland, and have shown a real possibility of creating flat fiber products with sensory properties containing carbon nanotubes. The presented research is a continuation of earlier work [25,26]. It shows the prototype of the Arduino embedded system as a proof of concept for counting the number of breaths by measuring the electrical resistance of the designed/elaborated printed textile sensor on the chest part of the garment. 

The essence of the work is the development of a T-shirt that enables the measurement of respiratory frequency together with a data collection system, and ensures user and sensory comfort (Total Hand Value). So far, research has been conducted on the production of breath sensors in the form of belts, bras and T-shirts, which did not provide user comfort due to the rigid elements installed in their structure. Studies related to printing have also been carried out, but their durability for maintenance processes and comfort of use, which has a significant impact on our feelings, were not verified in detail. The authors in that article presented different layouts of prints, such as curve, frame and square; a square was selected for the study. After a further series of tests, it was found that the curve also showed the correct results, and would certainly be better perceived by end users than the square. In addition, the authors in the previous article presented an analysis of the influence of humidity and temperature on the results. At that time, tests were also carried out using volunteers in a large-size climatic chamber (without the Arduino prototype).

Research on the biophysical and sensory chamber was carried out with the use of tools such as a skin model, an air permeability apparatus and the Kawabata Evaluation System. These tools allow one to objectively assess the usability of the developed solution and provide information on how the printout affects our feelings. 

## 2. Materials and Methods

### 2.1. Textile Material

As a textile material, a knitted fabric made of natural fibers with a left-right weave was selected. The choice was dictated by the fact that the final product, a T-shirt, should be skin-friendly and breathable, and not retain sweat against the skin. Considering the fact that the T-shirt was to collect information on the frequency of breathing, a knitwear characterized by a tight weave and containing elastomer fibers allowing for the reversible operation of the material was selected. The optimal textile material was selected on the basis of previous studies by the authors. The features of the knitted fabrics used in this study as the research material are presented in Table 1.

The following sample variants were tested:

- pure cotton knit
- cotton knitted fabric printed with carbon nanotube paste with the addition of polypyrrole

### 2.2. Printing Paste and Making Printouts

Carbon nanotubes and polypyrrole were selected as the printing material. This was primarily dictated by economic aspects related to the fact that carbon structures and conductive polymers are cheaper than gold or silver. In addition, previous research by the authors has proved that it is possible to obtain sensors with the desired properties that are durable in the processes of maintenance and use.

The printing composition was a mixture of the carbo nanotubes (AQUACYL ™ water dispersions) paste with a 0.5% admixture of Sigma Aldrich^®^ polypyrrole powder (CAS No. 30604-81-0). The article uses a combination of an ink composition containing multi-walled carbon nanotubes (MWCNTs) of the NC7000TM series with a diameter of 9.5 nm and a length of 1.5 μm in the amount of 3% by weight with 0.5% admixture of powdered polypyrrole by Sigma Aldrich^®^. Surfactants were added to improve stability. The dispersion itself was characterized by the following parameters: pH 7–11, boiling point 100 °C, melting point 0 °C, viscosity 100–200 cP. Based on the authors’ previous research, it was observed that the addition of 0.5% polypyrrole improved electrical conductivity by two orders of magnitude. In addition, a significant increase in the sensitivity of the tested sensors to deformation was observed in preliminary studies.

The printing of knitted fabrics was carried out with the use of a screen-printing machine MS-300FRO. It is a multifunctional device that allows you to make prints on various types of surfaces.

The knitted fabrics samples were stretched on the machine table in such a way as to allow them to be covered with the printing composition as precisely as possible. A net with a density of 43 mesh∙cm^−2^ was used (the holes in the printing mesh had a diameter of 76 μm).

For the printing paste, density, flow behavior index, viscosity and surface tension were determined, and the Ohnesorge number (Oh) and the 1/Oh factor were calculated. The diameter of the holes in the printing mesh included in the calculation was 76 μm.

According to previous work, it was determined the 1/Oh factor for the paste should be >~1.

### 2.3. Determination of the Surface Mass

The testing of the surface mass of samples of pure and printed knitted fabrics was carried out in accordance with the PN-EN 12127: 2000 Textiles standard. Flat textiles. Determination of mass per unit area using small samples.

Three samples of unprinted knitted fabrics and fabrics printed with nanotube paste with the addition of polypyrrole were prepared. Five samples with an area of 400 cm^2^ were cut from each variant. After 24-h acclimatization, the samples were weighed and their mass per unit area and standard deviation were calculated.

### 2.4. Determination of Thickness

The thickness of the knitted fabrics samples before and after printing was tested in accordance with the PN-EN ISO 5084: 1999 Textiles standard. Determination of the thickness of textiles.

After 24-h acclimatization, the thickness of each knitted variant was examined in ten different locations. The arithmetic mean and standard deviation were calculated from the obtained results.

### 2.5. Air Permeability

An air permeability assessment was carried out in accordance with the PN-EN ISO 9237: 1998 Textiles standard. Determination of air permeability of textiles.

To test the air permeability of the knitted fabrics samples, a specialized device of the FX 3300 type was used, which enables automatic and precise measurement. For each variant of the samples (unprinted, printed with carbon nanotube paste and printed with nanotube paste with the addition of polypyrrole), ten measurements were recorded, then the arithmetic mean and standard deviation were calculated from the obtained results.

### 2.6. Determination of Thermal Resistance

The evaluation of the thermal resistance of the knitted fabrics was carried out in accordance with the PN-EN ISO 11092: 2014 Textiles standard. Physiological properties. Measurement of thermal resistance and water vapor resistance under steady-state conditions (method of sweating heat-insulated plate).

The measuring instrument used in this study was a sweating thermally insulated plate placed in a chamber allowing control of the atmosphere. The apparatus simulates the process of heat exchange between the user and the environment, allowing the assessment of the thermal resistance of the material placed on the surface of the plate.

The samples were acclimated for at least 12 h under normal climate conditions. Then, the samples were placed in the measuring apparatus, which was set to the following parameters: plate temperature 35 °C, air temperature 20 °C, relative humidity 65%, air speed 1 m/s. Before taking the measurements, conditions inside the apparatus were given time to reach a state of equilibrium. The constant of the instrument necessary to calculate the thermal resistance of individual samples was also determined. After completing the measurements, the arithmetic mean and standard deviation of the thermal resistance were calculated for each variant of knitted fabrics.

### 2.7. Assessment of Sensory Comfort

The sensory comfort test of knitted fabrics before and after printing was carried out with the use of KES (Kawabata Evaluation System). This system makes it possible to evaluate the Total Hand Value (THV) based on measurable physical and mechanical properties of the garment materials. KES consists of four modules [27].

- KES-1—A module that evaluates material properties through unidirectional tension and shear. The tensile test is carried out until the tensile force is 500 N∙m^−1^ and the sample is relaxed. The KES-1 module first performs a shear test, then, using the same sample, it performs a tensile test [27].
- KES-2—A module that determines the properties of materials subjected to pure bending. The change in curvature during the bending test is constant and amounts to 0.5 cm^−1^∙s^−1^. During the bending test, the KES-2 module records changes in the bending moment as a function of curvature, producing a graph on the basis of which the following are calculated: bending stiffness related to the sample width and the value of the hysteresis loop width of the bending moment related to the sample width [27].
- KES-3—This module determines the properties of the tested materials subjected to compressive forces. The sample is transversely compressed with a piston at a speed of 0.02 mm∙s^−1^ until a compressive stress of 5 kN∙m^−2^ is achieved. During the test, the module records the course of the compression and relaxation curve, on the basis of which it is possible to evaluate the values characterizing the material during compression [27].
- KES-4—A module for assessing the surface properties of a material. The measurement of which is used to determine the kinetic coefficient of friction, and to assess the friction force. Additionally, the KES-4 module enables the measurement of the roughness of the material thanks to the foot, which, when the sample is shifted, moves over the unevenness of its surface [27].

The KN-403-KTU (SUMMER) mode was selected in the KES software, intended for testing women’s summer clothing.

### 2.8. Determination of Strength Properties

The strength properties of knitted fabrics samples were tested before printing in accordance with PN-EN ISO 13934-1: 2013 Textiles. Properties of flat products in stretching. Part 1: Determination of maximum force and relative elongation at maximum force by the strip method.

The study was carried out for the purpose of subsequent calculations of the unit pressure of knitted fabrics and the dimensions of the T-shirt in free state.

An INSTRON 5900 series testing machine was used for the test. Machine clamps were set at a distance of 100 mm, the beam speed was 100 mm∙min^−1^. 

### 2.9. Determination of Sensory Properties

The aim of the study was to check the influence of the mechanical stimulus on changes in the electrical resistance of knitted fabrics printed with conductive pastes. 

The measuring apparatus was an INSTRON 5900 series testing machine, a Keithley model 2000 digital multimeter and a connected computer with software recording resistance changes during the mechanical deformation of knitted fabrics. The deformation took place in ten cycles of tensioning and relaxing the knitted samples to the length of 5 mm, 10 mm, 15 mm and 20 mm. Based on the obtained results, the mean sensory factor was calculated, along with its standard deviation and the coefficient of variation for individual knitted fabrics variants.

### 2.10. Determination of Abrasion Resistance

The abrasion resistance of knitted fabrics coated with conductive pastes was assessed in accordance with the PN-EN ISO 12947-4: 2001 Textiles standard. Determination of abrasion resistance of flat products using the Martindale method. Assessment of the change in appearance.

The test was performed to evaluate the changes in the surface conductivity of the printed knitted fabrics before and after the functional test.

The measuring instrument used in this study was the Martindale apparatus used to carry out the abrasion test of flat textiles and the pilling test. 

### 2.11. Determination of Color Fastness

The color fastness test of knitted fabrics printed with conductive pastes was carried out in accordance with the PN-ISO 105-C06: 2010 Textiles standard. Color fastness tests. Part C06: Color fastness to domestic and communal washing.

The test was carried out to compare the electrically conductive properties of the prints before and after the functional test. The samples of the printed knitted fabrics with dimensions of 100 × 40 mm were washed for 30 min at the temperature of 40 °C. The composition of the detergent used is shown in Table 2.

At the end of washing, the samples were rinsed twice in separate portions of water for one minute, then excess water was drained from them and left hanging to dry.

### 2.12. Determination of Surface Conductivity

The surface conductivity of the printed knitted fabrics was assessed in accordance with PN-EN 1149-1: 2008 Protective clothing. Electrostatic properties. Part 1: Test method for surface resistivity.

Knitted fabrics with printed conductive pastes and samples after abrasion and washing were tested. The measuring apparatus was a system of electrodes consisting of a cylindrical and ring electrode coaxially arranged to each other and an ohmmeter connected to them. 

Additionally, a study of surface resistivity was performed using a Single Post Dielectric Resonator (SiPDR) called Microwave Q-Meter, manufactured by QWED. This apparatus allows for the non-contact measurement of the surface conductivity of samples of small thickness. 

### 2.13. Making a Personalized T-shirt

The final stage of the research work was to create a personalized T-shirt with a printed respiratory rhythm sensor and to conduct a pneumonia measurement.

Based on the results of the strength test of knitted fabrics and the dimensions of the model, the pressure exerted by the T-shirt was calculated in accordance with the La Place relationship and the dimensions of the product in its free state. Knowing the results of these calculations, it was possible to properly cut and sew the T-shirt. A conductive thread was sewn into the finished T-shirt, in the places where the printing edges were located, leading inside the side seams to the bottom edge of the T-shirt. Then, the ends of the threads were connected to the electrodes of a Keithley digital multimeter, and a computer connected to it, using appropriate software, recorded the changes in resistance caused by the movement of the chest of a model wearing a T-shirt. Based on the obtained results, the mean sensory coefficient and its standard deviation and coefficient of variation were calculated.

## 3. Arduino System for a Knitted Shirt with Stretchable Strain Sensors

**Figure 1:**
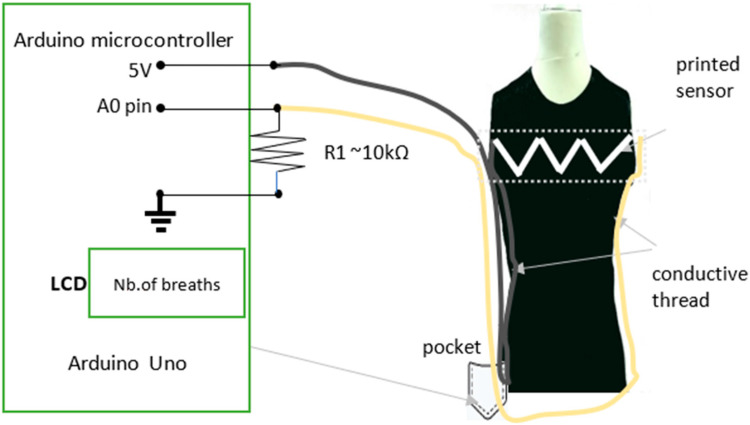
The microcontroller and textile sensor connection diagram (own elaboration).

The general idea of using Arduino in textiles is not new. In [28], researchers described the LilyPad Arduino, a fabric-based construction kit that enables novices to design and build their own soft wearables and other textile artifacts. An assortment of sensor and actuator elements can be sewn to cloth substrates and each other with conductive thread to build e-textiles. In [29], authors investigated aspects regarding the use of wearable electronic sensors, embedded in clothing for monitoring health using simple electronics such as the Arduino board to perform signal analysis processes. In [30], breathing rhythm and electrocardiography (ECG) were measured using flexible substrates on a T-shirt with bi-axial accelerometers involved. In [30], a similar proposal to the research presented in Figure 1 was presented; however, the model presented here is much easier to build and would be simpler to use in everyday life in its final production version. However, the model presented here is still not a mobile embedded system, but a first prototype of a working proof of concept. The authors do not recommend placing the future mobile embedded system at chest height, but instead suggest placing it in the side pocket at hip height, as shown in Figure 1, so the shape and size are comfortable for the user, which implies further work is needed on the mobile and miniaturized version.

**Figure 2:**
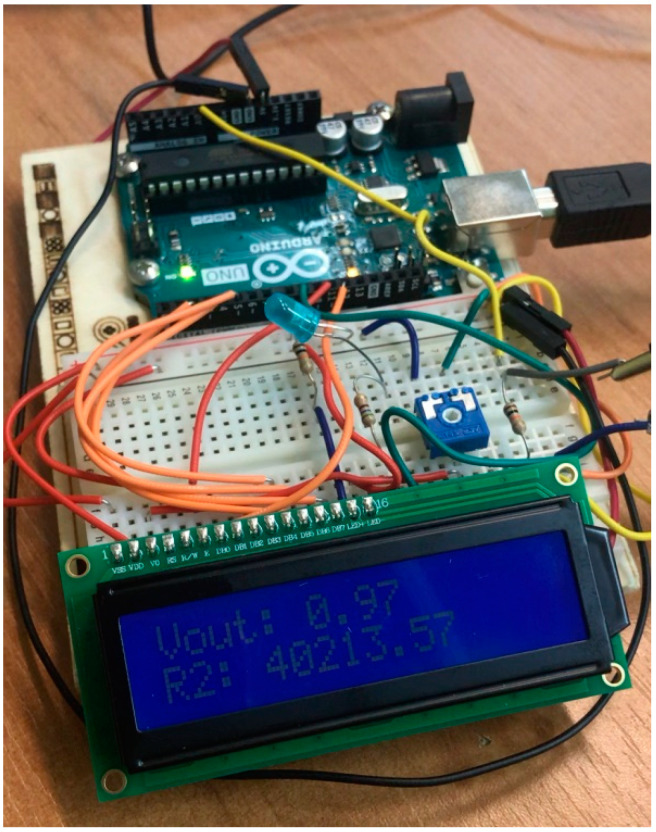
Photo of the Arduino microcontroller and cable connections to textile sensor.

In the presented textile sensor, the print was made on a black T-shirt with ink paste, which was also black. Such a procedure was aimed at making the sensor invisible to the user and their surroundings, and at the same time fulfilling the assumed function; therefore, it is marked with white lines in Figure 1. The solution presented in the article conducts data analysis and reports human live parameters with the Arduino embedded system as a proof of concept for innovative solutions in health care. The Arduino embedded system prototype is shown in Figure 2.

### 3.1. Practical Measurements

- Research apparatus
  - Arduino or Genuino Board connected to measuring electrodes in Figure 2,
  - T-shirt with print containing sensors receiving and transmitting information about the tested subject in Figure 1 [4].
- Idea of research

**Figure 3:**
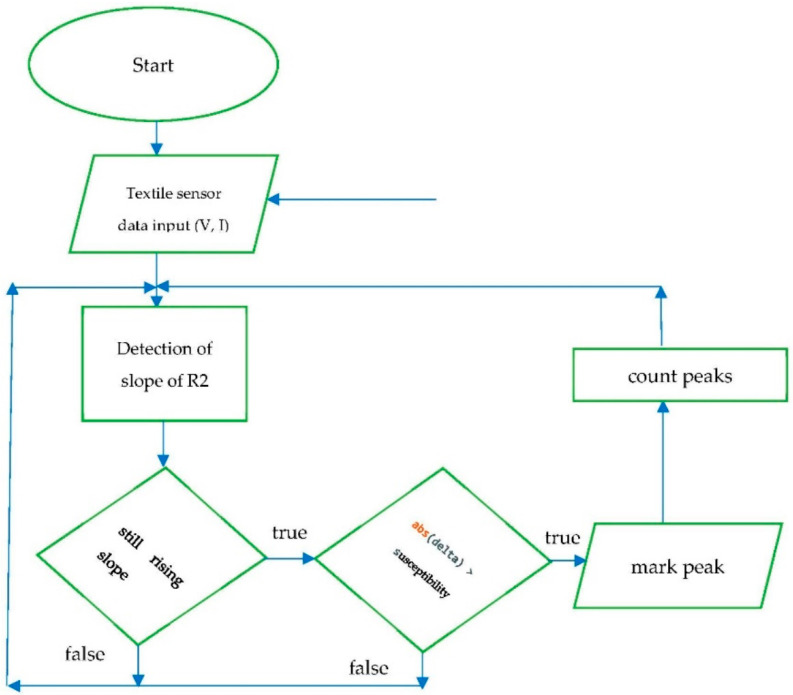
Block diagram of algorithm.

The idea of this study was the continuation of the research shown in [26], and to measure the electrical resistance of the Arduino microcontroller of an elaborated textile sensor to track changes in electrical resistance during the normal activity of a person who is wearing the T-shirt. The idea behind the application of strain sensors with data analysis to report on human live parameters with the Arduino embedded system was a very important element aimed at developing new and innovative solutions for health care. The number of breaths is calculated by a logical algorithm inside Arduino by counting the picks of resistance within 60 s. An exemplary architecture diagram is shown in Figure 3. Two resistors were used: the first resistor was ca. 10 kΩ, and the second was represented by the textile sensor. From the point of view of Ohm’s law, it is important that the resistors were connected in series. The resistor with a known resistance of ca. 10 kΩ between the ground and the cable was connected to the A_0_ pin, while the tested textile sensor resistor was between the cable connected to the A_0_ pin and 5V from Arduino.

According to Ohm’s law, the current flowing in the system is the quotient of voltage and the resistance of a given system (1).

I = U∙R(1)

where: I—current, U—voltage, R—resistance.

The presented system consists of two resistors connected in series with values denoted as R1 and R2. R1 is the known resistor, while R2 is the textile sensor. Current I flows through the system. Such current flows through both R1 and R2. The voltage drop occurs in the whole system and, in each of the resistors, the voltage proportionally changes to its value. The voltage that falls in the whole system is the voltage taken from Arduino, i.e., 5V—denoted as U, the voltage in R1 is denoted as U1. Having the above in mind, we can determine the following Formula (2):

U/(R1 + R2) = U1/R1(2)

R2 = ((R1 × U)/U1) − R1 (3)

where,

R1—known resistor 10 kΩ, R2—resistance of the textile sensor.

Formula (3) is calculated inside the microcontroller. The values are computed and analyzed in Arduino. A value of peaks per minute, corresponding to the number of human breaths, is calculated and displayed on the LCD screen. Through normal breathing, the textile sensor electrical resistance is changed; thus, we change the value of resistance, which is connected to the center pin of the resistor series. This changes the voltage at the center pin. This voltage is the analog voltage that is read as an input by the Analog-to-Digital Converter (ADC).

### 3.2. Explanation of Arduinio Program

The program listed in Appendix A includes the LCD library. Lines 3:23 are declarations of variables; including the textile sensor seen as the resistance connected to the analog input A0. In line 10, there is declaration of the LCD screen. The numbers are the port numbers that are connected to the pins on the LCD from left to right. In lines 15 and 16, breaths per minute and an incremental counter vars are shown. In line 19, there is a delta variable that has the values of the difference between the previous sensor resistance and the currently calculated one. In line 20, there is a variable concerning the sensitivity of the algorithm to detect changes in the sensor resistance. The variable is initialized as 80 kΩ. Two resistors are used: the first resistor is ca. 10 kΩ and is represented by variable R1, and the second is represented by the variable R2, which is the textile sensor.

In lines 33:35, there is a calculation of R2 for every cycle and a detection of slope of the R2 resistance of a textile sensor (algorithm diagram at Figure 3.)

The sign of the delta variable in lines 44:52 informs whether there is an increase in resistance (delta <= 0) or a decrease (delta > 0); i.e., whether there is an inhalation or exhalation of air from the lungs. A susceptibility variable is used to determine the peak designation and is responsible for the stability of the algorithm. That means if the abs(delta) is big enough in increasing the slope trend of sensor resistance, then this marks a peak.

The algorithm works in lines 53:89 in such a way that the last measurement shows the increasing direction of the sensor resistance, and the current measurement informs that the increase is over; i.e., if the resistance does not increase more, it means that we obtained a peak and the value of the delta variable increases and is then marked as the peak.

## 4. Results

### 4.1. The Surface Mass of Knitted Fabrics

After weighing the samples of knitted fabrics made in accordance with the methodology described in Section 2.3, their mass per unit area was calculated in accordance with the formula (4), and the arithmetic mean of the area masses was calculated.

(4) $$M_{p}=\frac{m}{L_{a}\cdot S_{a}}$$

where: *M*_p_—product surface mass, g∙m^−2^

*m*—mass of acclimatized samples, g

*S_a_*—width of the acclimatized sample, m

*L_a_*—length of the acclimatized sample, m

Table 3 presents the values of the average area weight and standard deviations of the area weight for individual variants of samples.

### 4.2. Thickness

The knitted fabrics thickness was assessed in accordance with the methodology described in Section 2.4. Table 4 shows the values of the mean thickness and the standard deviation of the thickness for individual samples.

### 4.3. Air Permeability

The air permeability test was carried out in accordance with the methodology described in Section 2.5. The apparatus calculated the velocity of the air passing through the sample according to formula (5).

(5) $$R=\frac{{\bar{q}}_{v}}{A}\times 167$$

where: *R*—air permeability, mm∙s^−1^

${\bar{q}}_{v}$—arithmetic mean of the amount of flowing air, dm^3^∙min^−1^

*A*—tested surface of the product, cm^2^

167—conversion factor

Table 5 presents the values of the average air permeability and its standard deviations for individual variants of knitted fabrics.

### 4.4. Thermal Resistance

The thermal resistance test was carried out in accordance with the methodology provided in Section 2.6. The software of the computer connected with the measuring apparatus calculated the thermal resistance of individual knitted fabrics based on the formula (6).

(6) $$R_{ct}=\frac{(T_{m}-T_{a})\cdot A\;}{H-\Delta H_{c}}-R_{ct0}$$

where: *R_ct_*—thermal resistance, m^2^∙K∙W^−1^

*T_m_*—temperature of the measuring plate, °C

*T_a_*—air temperature in the measuring chamber, °C

*A*—area of the measuring plate, m^2^

*H*—heating power supplied to the measuring plate, W

Δ*H_c_*—heating power correction

*R*_*ct*0_—thermal resistance of an uninsulated panel, m^2^∙K∙W^−1^

Table 6 presents average values of thermal resistance and its standard deviation for individual knitted fabrics.

### 4.5. Sensory Comfort

The sensory comfort test was carried out in accordance with the methodology described in Section 2.7. Table 7 presents the numerical values of the grip characteristics of all knitted fabrics.

### 4.6. Strength Properties

After testing the knitted fabrics before printing in accordance with the methodology described in Section 2.8, the arithmetic mean of the maximum force and the relative elongation at maximum force were calculated. Table 8 presents the values of the maximum force and relative elongation at maximum force and their standard deviations.

### 4.7. Sensory Properties

The evaluation of the sensory properties of the printed knitted fabrics was carried out in accordance with the methodology described in Section 2.9. The sensory coefficient for the individual cycles of stressing and relaxing the samples was calculated according to the formula (7), Table 9:

(7) $$R_{rel}=\frac{R-R_{0}}{R_{0}}\times 100$$

where: *R_rel_*—sensory factor, %

*R*_0_—single cycle initial resistance, Ω

*R*—final resistance in a single cycle, Ω

**Table 9:** Values of sensory coefficients, standard deviation and coefficients of variability of sensory properties of individual variants of knitted fabrics.

Type of Sample	Tension Distance, mm	Sensory Coefficient, %	Standard Deviation, %	Coefficient of Variation
Cotton + MWCNT + PPY	5	27.49	3.13	0.11
	10	61.93	10.91	0.18
	15	89.89	12.04	0.13
	20	136.91	5.72	0.04

### 4.8. Surface Conductivity

The assessment of the conductive properties of prints was carried out in accordance with the methodology described in Section 2.12. The test covered samples of knitted fabrics after printing and samples subjected to functional tests (washing and abrasion), carried out in accordance with the methodology described in Section 2.10 and Section 2.11. Table 10 and Table 11 shows the values of the average electrical resistance and its standard deviation for individual samples, tested with standardized measuring equipment.

According to the obtained results, it was found that the samples not subjected to operational tests had the best surface conductivity. The samples showed a deterioration of electrically conductive properties after operational tests, where the electric resistance appeared to be higher in the case of samples subjected to the washing process.

### 4.9. Practical Measurement and Aim of Calculations

#### The Instron Using the Arduino Program

**Figure 4:**
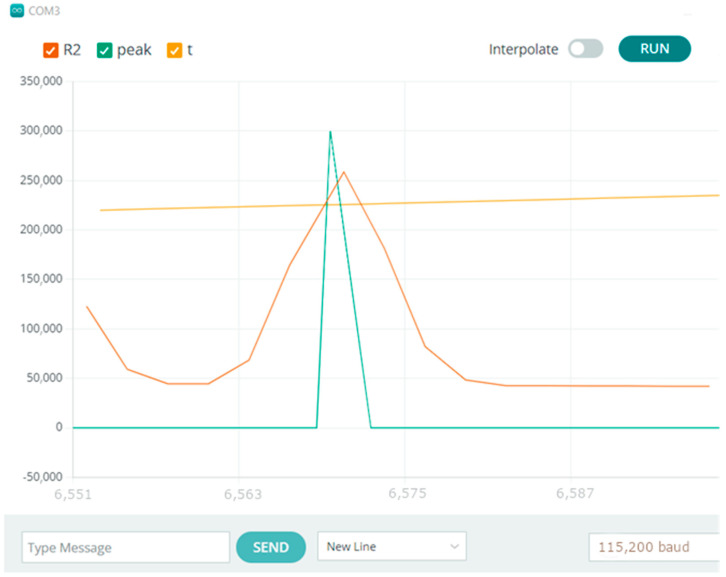
Arduino serial plot peak recognition.

At first, the sensitivity had to be adjusted to match the physical characteristics of the printed sensor. In the screenshot from the Arduino serial plot in Figure 4, we can see the absolute value from the delta variable and the peak of resistance detected by the algorithm by setting the variable peak with an arbitrary value informing about the detection of breath.

Assuming that the distance between the clamps will be 10 cm, the stretching distance is 10 mm; i.e., if the stretch is by 10%, it is suggested to maneuver the speed of the beam.

For simulation:

- slow breathing—Bradypnea—beam speed 160 mm/min (eight breaths per min)
- normal breathing—beam speed 240 mm/min (12 breaths per min)
- increased breathing—Hyperpnea—beam speed 400 mm/min (20 breaths per min)
- increased breathing—Tachypnea—beam speed 480 mm/min (24 breaths per min)

**Figure 5:**
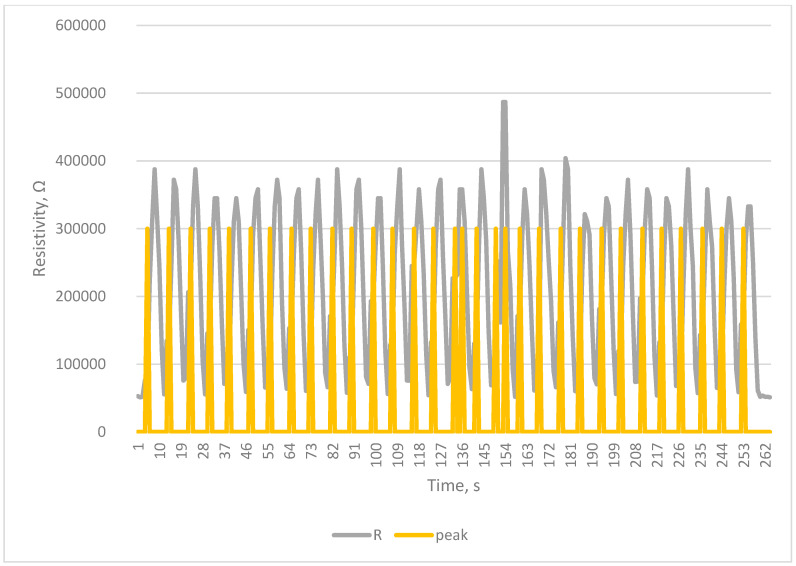
Detection of breath in simulation of slow breathing—Bradypnea—eight breaths per min.

**Figure 6:**
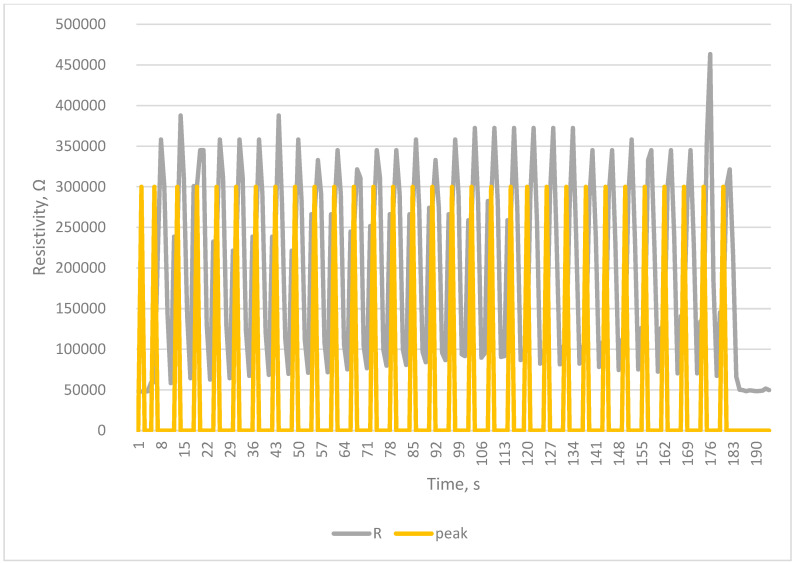
Detection of breath in simulation of normal breathing—12 breaths per min.

**Figure 7:**
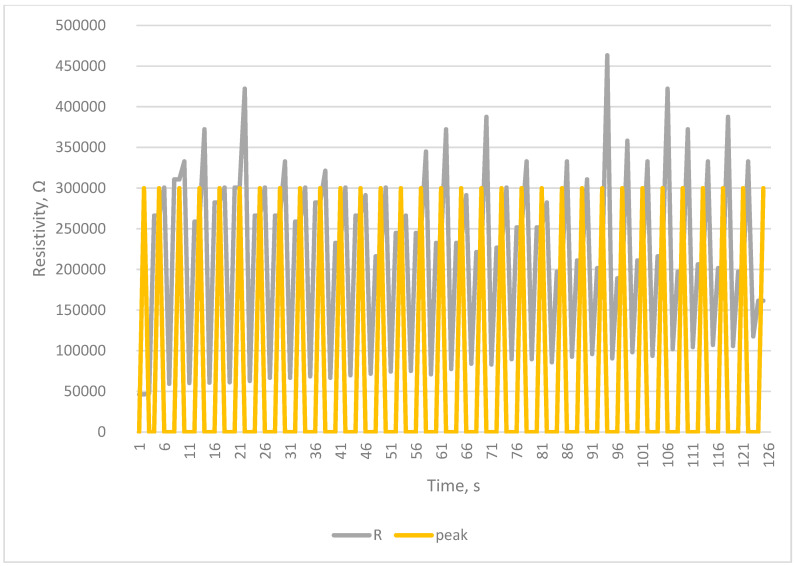
Detection of breath in simulation of increased breathing—Hyperpnea—20 breaths per min.

**Figure 8:**
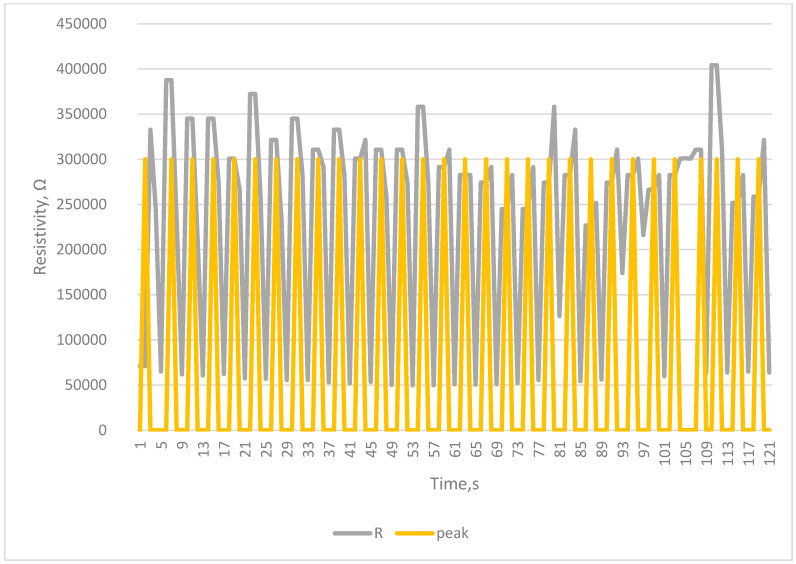
Detection of breath in simulation of increased breathing—Tachypnea—24 breaths per min.

The tests carried out using a testing machine as a breath simulator confirm the correctness of the collected signals and the correctness of counting breaths by the developed system Figure 5, Figure 6, Figure 7 and Figure 8.

**Figure 9:**
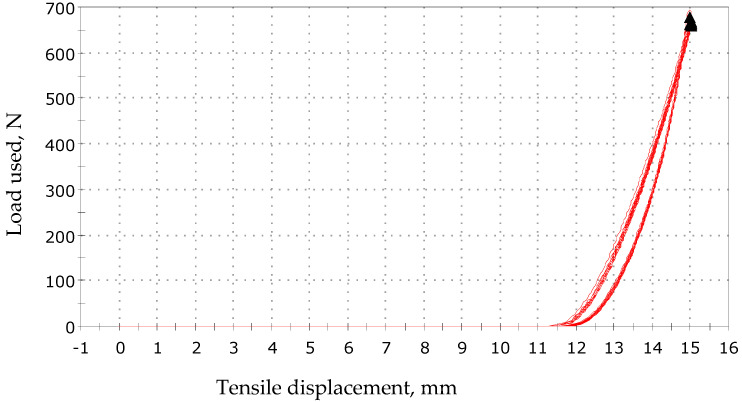
Tensile displacement by load 0–700 N.

**Figure 10:**
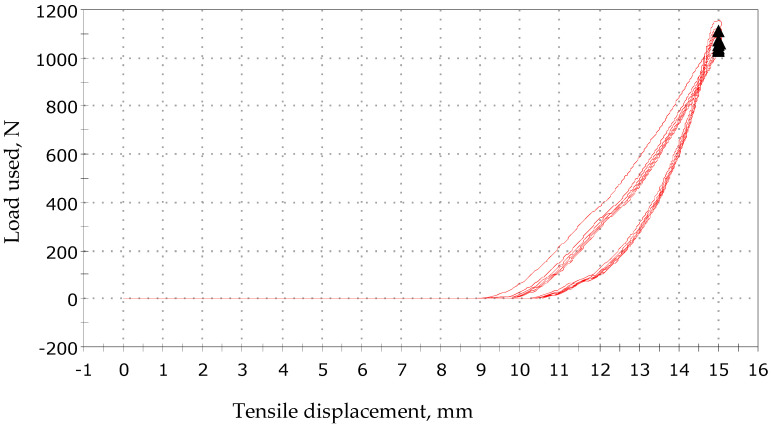
Tensile displacement by load 0–1200 N.

**Figure 11:**
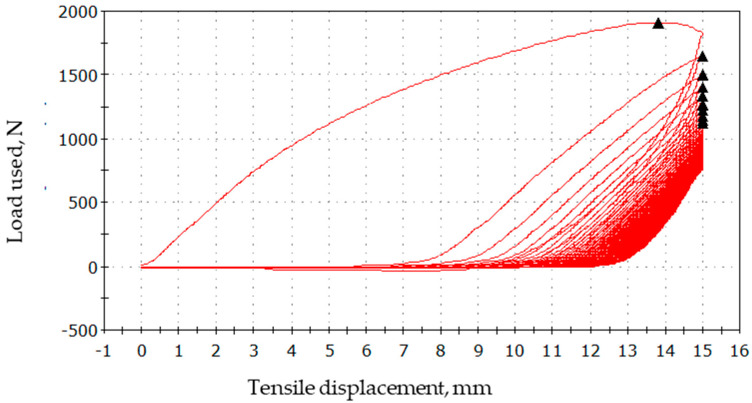
Tensile displacement by load 0–2000 N.

In addition, hysteresis loops for various loads were measured for the textile sensor. The presented graphs in Figure 9, Figure 10 and Figure 11 show that the samples operate in a cyclical manner without any particular disturbances.

As part of the previous work carried out by the authors, a procedure was developed that included a suitably modified exercise test, which was carried out in accordance with the Bruce protocol, in which the body’s ability to undertake physical effort (efficiency) is determined. The procedure assumes that, for the same person, the measure of the ergonomics of different clothes will be the maximum physical effort that the tested person will be able to take during the test in different clothes (the better the clothes, the greater the effort that can be made in them).

The study of the T-shirts developed and presented in the article was carried out in accordance with a test specially modified for this purpose, which measured the maximum effort that the tested person was able to make using a treadmill. The result of the research was the highest value of the effort made by the subject.

The test results were very similar to the reference sensor used in the medical diagnostics laboratory (Table 12).

The obtained results allow us to conclude that the developed system allows for the correct recording of the respiratory rate.

## 5. Discussion of Research Results

The thermal resistance after covering the knitted fabric samples with prints increased. However, the scale of changes in thermal resistance was slightly higher, which may indicate that the printing surface was properly selected so as not to adversely affect the functional properties of the developed product in the form of a T-shirt.

In the case of the assessment of total grip, its increase was noted after applying prints to the surface of knitted fabrics. This means that the authors managed to slightly improve the sensory sensations associated with the grip in relation to the non-printed material.

For the research in this work, the authors selected an ink that had better sensory properties than in their earlier works. It can be observed that the sensory sensitivity increased with the increase in the percentage of deformability of the tested T-shirt, and so, for example, for a distance of 5 mm, the sensory sensitivity was 27.49%, and for 15 mm it was 89.89%.

In the case of surface conductivity measurements using the standardized method, the samples printed with the paste with the addition of polypyrrole were characterized by higher resistance values than the measurement of surface conductivity using the non-normalized method. Knitted fabric samples subjected to an abrasion test gave inconclusive results. The knitted fabric showed an improvement in surface conductivity tested by the standardized method, while the non-normalized method showed a deterioration in conductivity. This may occur due to the fact that, during the rubbing process, some of the abraded printing material was rubbed into other places, which filled the gaps and improved conductivity. In the case of samples after the washing process, the conductivity slightly deteriorated.

The conducted research confirms the possibility of recording the frequency of breathing both in healthy people and in people with respiratory disorders. The developed measurement system in the form of a shirt together with the embedded system will enable medical specialists to monitor abnormal respiratory rates even at home. The production implementation, if any, will require the application of better methods of resistance measurement with less power consumption than a used voltage divider, and a mobile and miniaturized version of it. 

The developed breathing sensor will enable the registration of disorders such as:

- Bradypnea is a medical term used to describe abnormally slow breathing (8–10 breaths per minute),
- Tachypnea is the medical term used to describe an elevated respiratory rate. This rapid respiratory rate is usually shallow compared to hyperpnea, which can be rapid and profound (greater than 24 breaths per minute),
- Hyperpnea refers to breathing that is abnormally deep and feels laborious. May occur with or without rapid breathing (about 20 breaths per minute)
- Apnea literally means “no breath” and refers to the absence of breathing (about two breaths per minute).

The developed system also allows the breathing of children to be monitored, who have a faster respiratory rate than adults, and the “normal” respiratory rate can significantly vary with age. Normal respiratory rate ranges for children of different ages include:

Newborn: 30–60 breaths per minute
Infant (1 to 12 months): 30–60 breaths per minute
Toddler (1–2 years): 24–40 breaths per minute
Preschooler (3–5 years): 22–34 breaths per minute
School age child (6–12 years old): 18–30 breaths per minute
Adolescents (13–17 years): 12–16 breaths per minute
The average respiratory rate in a healthy adult is 12 to 16 breaths per minute.

The Arduino-based system was proven to work, as seen in Figure 4, Figure 5, Figure 6, Figure 7 and Figure 8. Breaths simulated on the Instron were accurately detected.

## 6. Conclusions

The screen printing technique is an excellent technological solution for creating sensors for pneumography measurements. The analysis of the research presented in the article allows us to conclude that the methods of screen printing for the production of textile resistance sensors allow for the quick and non-invasive production of smart textiles useful in monitoring vital signs. However, it should be remembered that applying printing pastes to textile surfaces reduces air permeability and increases thermal resistance. Therefore, surfaces related to the shape and size of the sensor should be carefully designed so as not to adversely affect the biophysical comfort of the end user. By analyzing sensory comfort tests, it was observed that, after applying prints to the surface of knitted fabrics, their total hand (THV) increases. This type of behavior was influenced by the fact that, after printing, the parameters related to bending stiffness, brittleness and filling improved.

The conducted research allows for the conclusion that the sensory coefficient linearly increases with stretching distance.

A very important feature of sensors prepared in this way is that they can undergo relatively large elastic deformations. Both bending and stretching of the textile substrate do not negatively affect the signal transmitted by the sensor. In addition, sensors of this type can continuously work without compromising the accuracy of the transferred data. The conducted analysis of the tests allows the conclusion that the quality of the transmitted signal is not significantly affected by the shape of the printout, which is confirmed by the fact that printouts in the shape of rectangles were made for tests simulated with a machine; whereas for T-shirts, this was in the shape of an ECG signal. 

However, it should be remembered that smart devices used to monitor health should be personalized in such a way that they closely adhere to the body and do not cause excessive pressure on the chest.

The analysis of the research also highlighted the existing imperfections associated with the everyday use of clothing. Textile products, unlike rigid electronic systems currently used in healthcare, are reusable items, exposed to friction processes during use and require maintenance (washing). Therefore, they are subject to higher requirements than disposable electrodes. Based on the conducted research, it can be concluded that more detailed research should be carried out related to the combination of printing compositions with textile substrates. This is related to the fact that polymer raw materials have different surface tension, which is directly related to permanent connections between the substrate and the print.

In addition, the conducted research highlighted one more problem that still occurs even in hospital systems, namely the correct counting of the respiratory rate at rest. When the user moves, there are additional signals related to the movement of the hands, which makes it possible to detect additional “false” breaths that are not actually there. Therefore, in their subsequent works, the authors will focus on optimizing the location of the breath sensor and refining the design of the shirt, in which the occurrence of undesirable artifacts will be minimized.

## Figures and Tables

**Table 1:** Characteristics of the research material. Own elaboration.

Name	Composition	Weave
Cotton knit	86% cotton, 14% lycra	Left right

**Table 2:** Composition of the washing agent used in the test. Source: Own elaboration.

Composition	Mass Share, %
Sodium alkyl sulfonate, linear (LAS)	14.00 ± 0.02
Ethoxylated alcohol	2.30 ± 0.02
Soap with high molecular weight	2.50 ± 0.02
Sodium tripolyphosphate	48.00 ± 0.02
Sodium silicate (SiO_2_/Na_2_O = 2/1)	9.70 ± 0.02
Sodium sulphate	15.40 ± 0.02
Carboxy-methyl cellulose (CMC)	0.25 ± 0.02
Water	7.85 ± 0.02

**Table 3:** Average surface mass values and their standard deviations for individual samples.

Name	Surface Mass, g∙m^−2^	Standard Deviation, g∙m^−2^
Cotton	315.23	0.55
Cotton + MWCNT + PPY	347.88	0.20

**Table 4:** Average thickness values and their standard deviations for individual samples.

Name	Thickness, mm	Standard Deviation, mm
Cotton	0.85	0.03
Cotton + MWCNT + PPY	0.90	0.01

**Table 5:** Values of the average air permeability and its standard deviations for knitted fabrics.

Name	Air Permeability, mm∙s^−1^	Standard Deviation, mm∙s^−1^
Cotton	68.80	1.53
Cotton + MWCNT + PPY	54.78	3.15

**Table 6:** Average values of thermal resistance and its standard deviation for individual knitted fabrics.

Name	Thermal Resistance, m^2^∙K∙W^−1^	Standard Deviation, m^2^∙K∙W^−1^
Cotton	0.0236	0.0014
Cotton + MWCNT + PPY	0.0321	0.0000

**Table 7:** Values of individual grip characteristics of the tested knitted fabrics.

Sample Name	THV	Koshi	Fukurami	Shari
Cotton	2.74	11.59	13.14	0.40
Cotton + MWCNT + PPY	3.60	14.48	13.88	2.60

**Table 8:** Maximum force and elongation results at maximum force with standard deviations.

Sample Name	Maximum Force, N	Standard Deviation of the Maximum Force, N	Relative Elongation, %	Standard Deviation of the Relative Elongation, %
Cotton	150.88	5.62	228.90	3.94

**Table 10:** Results of average resistance values and their standard deviations for individual knitted fabrics tested using the standardized method.

			Type of Sample	
Sample Name	Calculated Value	After Printing	After Abrasion	After Washing
Cotton + MWCNT + PPY	average resistance, kΩ	6.06	4.63	6.28
	standard deviation, kΩ	17.90	16.39	36.27

**Table 11:** The values of the average resistance and its standard deviation of individual samples tested using the non-standard method.

			Type of Sample	
Sample Name	Calculated Value	After Printing	After Abrasion	After Washing
Cotton + MWCNT + PPY	average resistance, kΩ	0.71	2.51	3.82
	standard deviation, kΩ	0.40	0.87	0.55

**Table 12:** Respiratory rate monitoring test results.

Sample Name	MET (Metabolic Equivalent of Task)	Developed Shirt	Reference System
Cotton + MWCNT + PPY	4.8	15	13
	6.8	20	21
	9.6	30	27
	13.5	40	40
	16.1	56	55
	19.8	58	59

## Data Availability

Not applicable.

## Appendix A

Source code of Arduino Uno program for detecting the breaths in textile sensor.

1: #include <LiquidCrystal.h> 2: /* ******************* Project ************************ J.Wojciechowski, E.Skrzetuska “The Use of the Arduino Embedded System as a Prototype of a Mobile System Controlling a Person’s Breathing Using a Sensor Printed on a T-shirt” ********************************************************** */ 3: const inttextilesensor = A0; 4: intVin =5; 5: intraw =0; 6: floatVout =0; 7: floatbuffer =0; 8: floatR1 =9700; 9: floatR2 =0; 10:LiquidCrystallcd(12,11,5,4,3,2); 11: floatmaxVal =0; 12: uint32_tlastTimeSample =0; 13: uint32_tlastTimeSampleperminute =0; 14: boolinPeak =false; 15: intcountperminute =0; 16: intcontinuouscount =0; 17: boolinRisingSlope =false; 18: boolinDescendingSlope =false; 19: floatdelta =0; 20: floatsusceptibility =80000; 21: boollatch =false; 22: intlastDirection = -1; 23: boolidle_flag =false; 24: voidsetup() { 25:lcd.begin(16,2); 26:lcd.clear(); 27:Serial.begin(115200); 28:} 29: voidloop() { 30:lcd.setCursor(0,0); 31:raw =analogRead(textilesensor); 32:if(raw) { 33: buffer = raw * Vin; 34: Vout =(buffer)/1024.0; 35: buffer =(Vin / Vout)-1; 36: R2 = R1 * buffer; 37: lcd.print(“Vout: “); 38: lcd.print(Vout); 39: lcd.setCursor(0,1); 40: lcd.print(“R2: “); 41: lcd.print(R2); 42: lastTimeSample =millis(); 43: delta = maxVal - R2; 44: if(delta <=0&&abs(delta)> susceptibility) { 45: idle_flag =false; 46: inDescendingSlope =false; 47: inRisingSlope =true; 48: }else if(delta >0&&abs(delta)> susceptibility) { 49: idle_flag =false; 50: inDescendingSlope =true; 51: inRisingSlope =false; 52: } 53: if(idle_flag ==false) { 54: if(lastDirection ==1&& latch ==true&& inRisingSlope ==false) { 55: latch =false; 56: } 57: if(inRisingSlope ==true&& inDescendingSlope ==false&& latch ==false) { 58: countperminute++; 59: continuouscount++; 60: inPeak =true; 61: Serial.println(“peak”); 62: Serial.print(“countperminute: “); 63: Serial.println(countperminute); 64: Serial.print(“continuouscount. “); 65: Serial.println(continuouscount); 66: latch =true; 67: }else{ 68: inPeak =false; 69: } 70: if(millis()- lastTimeSampleperminute >60000) {// Every 1min: 71: lastTimeSampleperminute =millis(); 72: countperminute =0; 73: } 74: if(inRisingSlope) { 75: lastDirection =1; 76: } 77: if(inDescendingSlope) { 78: lastDirection =0; 79: } 80: } 81: maxVal =0; 82: delta =0; 83: inRisingSlope =false; 84: inDescendingSlope =false; 85: if(R2 > maxVal) { 86: maxVal = R2; 87: } 88: delay(1000); 89:}}
